# Wine Flavonoids in Health and Disease Prevention

**DOI:** 10.3390/molecules22020292

**Published:** 2017-02-14

**Authors:** Iva Fernandes, Rosa Pérez-Gregorio, Susana Soares, Nuno Mateus, Victor de Freitas

**Affiliations:** LAQV/REQUIMTE, Departamento de Química e Bioquímica, Faculdade de Ciências da Universidade do Porto, Rua do Campo Alegre 687, 4169-007 Porto, Portugal; iva.fernandes@fc.up.pt (I.F.); rosi.perez.gregorio@gmail.com (R.P.-G.); susana.soares@fc.up.pt (S.S.); vfreitas@fc.up.pt (V.d.F.)

**Keywords:** wine polyphenols, polyphenol-protein interactions, bioavailability of wine polyphenols, health, biological effects

## Abstract

Wine, and particularly red wine, is a beverage with a great chemical complexity that is in continuous evolution. Chemically, wine is a hydroalcoholic solution (~78% water) that comprises a wide variety of chemical components, including aldehydes, esters, ketones, lipids, minerals, organic acids, phenolics, soluble proteins, sugars and vitamins. Flavonoids constitute a major group of polyphenolic compounds which are directly associated with the organoleptic and health-promoting properties of red wine. However, due to the insufficient epidemiological and in vivo evidences on this subject, the presence of a high number of variables such as human age, metabolism, the presence of alcohol, the complex wine chemistry, and the wide array of in vivo biological effects of these compounds suggest that only cautious conclusions may be drawn from studies focusing on the direct effect of wine and any specific health issue. Nevertheless, there are several reports on the health protective properties of wine phenolics for several diseases such as cardiovascular diseases, some cancers, obesity, neurodegenerative diseases, diabetes, allergies and osteoporosis. The different interactions that wine flavonoids may have with key biological targets are crucial for some of these health-promoting effects. The interaction between some wine flavonoids and some specific enzymes are one example. The way wine flavonoids may be absorbed and metabolized could interfere with their bioavailability and therefore in their health-promoting effect. Hence, some reports have focused on flavonoids absorption, metabolism, microbiota effect and overall on flavonoids bioavailability. This review summarizes some of these major issues which are directly related to the potential health-promoting effects of wine flavonoids. Reports related to flavonoids and health highlight some relevant scientific information. However, there is still a gap between the knowledge of wine flavonoids bioavailability and their health-promoting effects. More in vivo results as well as studies focused on flavonoid metabolites are still required. Moreover, it is also necessary to better understand how biological interactions (with microbiota and cells, enzymes or general biological systems) could interfere with flavonoid bioavailability.

## 1. Introduction

Wine is a dietary source of phytochemicals. Among these, red wine phenolics are commonly divided into two major groups: flavonoids and non-flavonoids. The main flavonoid compounds present in red wine comprise several classes such as flavanols [(epi)catechin], flavonols (e.g., myricetin and quercetin) and anthocyanins (e.g., malvidin-3-glucoside), while non-flavonoid compounds present in wine include phenolic acids, phenols and stilbenes.

Many years ago, the moderate daily consumption of red wine was proposed as a contributing factor to the observed lower incidence of coronary heart disease in France despite the ingestion of high levels of saturated fat [1]. Since then, several epidemiological studies have shown a positive association between red wine ingestion and human health. In fact, epidemiological studies from diverse populations have revealed that individuals who usually consume moderate amounts of wine experience a 20% to 30% reduction in all-cause mortality, particularly cardiovascular mortality [2,3].

Red wine is different from other alcoholic beverages due to its content in various phenolic compounds. It is known that excessive alcohol consumption increases the risk of liver cirrhosis and cancers, mostly those of the upper digestive and respiratory tract, while low to moderate red wine consumption has been associated to health-promoting properties [4].

Many reported health-promoting activities associated to red wine consumption have been positively correlated with their flavonoid compounds composition [5,6,7]. However, due to the lack of more of epidemiological and in vivo studies, the presence of a high number of variables such as human age, interindividual and gender differences, metabolism, microbiota, the complex wine chemistry, and the wide array of in vivo biological effects of these compounds suggests that only cautious conclusions may be drawn from studies on red wine and a specific health issue.

Nowadays, the knowledge of red wine phenolic composition has increased considerably [8,9,10] and numerous scientific studies have revealed the in vitro biological properties of some red wine flavonoids. The prevention of some diseases is related to the strong antioxidant capacity of these compounds. Through their antioxidant property, flavonoids could potentially prevent free-radical-related injury. They are able to scavenge a wide range of reactive oxygen (ROS), nitrogen (NO) and chlorine species [11,12,13] as well as inhibit the production of reactive species [13,14].

However, another emerging concept is that the activity of flavonoids against several diseases is associated to much more complex effects related to cell proteins’ interactions and cell signaling [15,16]. It has been proposed that flavonoids exert their effects through their interaction (and thus inhibition and modulation activities) with a wide range of receptors, enzymes and even transcription molecules.

In the case of red wine tannins (a group of polyphenols known for their high ability to interact/precipitate proteins), the interaction with biological proteins is at the origin of their positive or negative biological and health effects. In addition, these interactions could compromise tannins’ absorption, metabolism and their bioactivities.

Although the study of these biological interactions has been the area of research of numerous groups and the literature has provided great advances on understanding their impact on human health, the mechanisms by which biological events lead to disease prevention are still a deep area of research.

In fact, it seems that there is a great gap between in vitro and in vivo studies. Some questions arise: which compounds are actually involved in the biological events? Which amounts of these compounds are required? Indeed, it is currently acknowledged that the bioactive forms of flavonoids in vivo are not necessarily the same forms that occur in food.

There is now strong evidence that the molecules responsible for those effects are probably not the ingested ones but rather their metabolites that occur after absorption or after the action of microbiota. The identification and quantification of these metabolites has not been an easy task but improvement of analytical methods and sensitivity has allowed some advances in this area. Methylation, sulfation and glucuronidation as a result of flavonoid biotransformation will lead to diminished antioxidant capacity. This discovery confers no physiological relevance to many of the in vitro and in vivo studies performed with the original molecules and also to studies in which the concentration ranges of the compounds tested are above the ranges detected in human plasma, urine and faecal samples.

But, in the end, other works have found that anthocyanin methylated metabolites still retain significant radical scavenging activity. The conjugation with methyl groups decreased or did not alter the antiproliferative effect of the original anthocyanin [17].

Due to the paucity of human studies concerning red wine bioavailability and biological interactions, this review will include some data using the main red wine flavonoids as purified compounds or as included in other food matrices.

**Figure 1:**
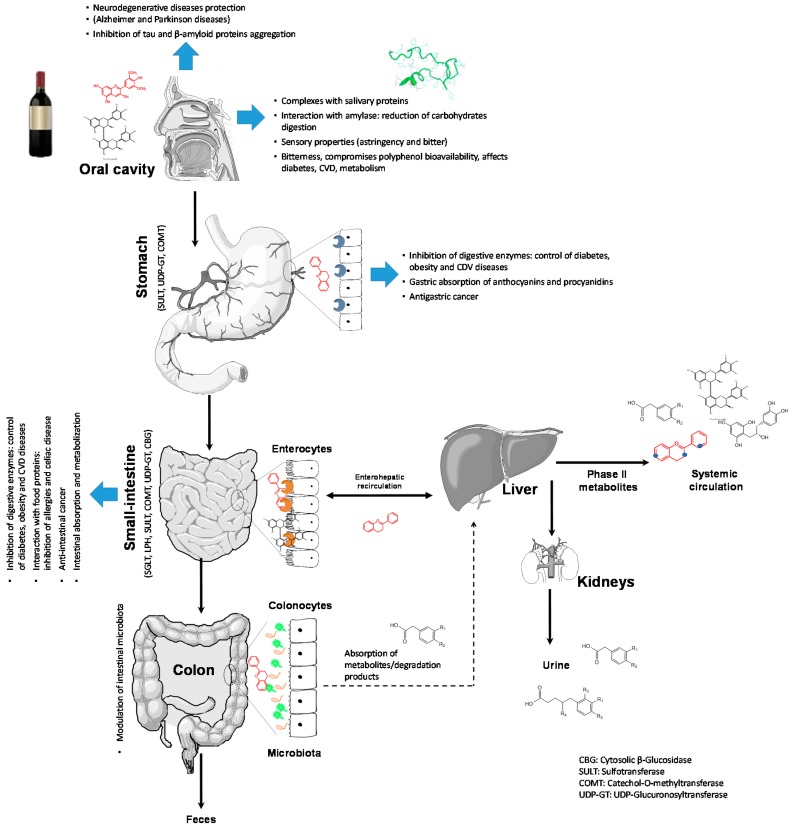
Schematic representation of the journey of red wine components after being ingested. They must pass through the oral cavity, the gastrointestinal tract, undergo metabolic events, pass cellular barriers and eventually trigger a biological action. The text summarizes the most relevant biological activities attributed to polyphenols in the different organs. The schemes highlight some of the major activities in the gastrointestinal tract.

When consuming red wine, there is a long journey before its components can exert a health-promoting effect. They must pass through the oral cavity, the gastrointestinal tract, suffer the action of microbiota, undergo metabolic reactions, pass cellular barriers, and eventually trigger a biological event (Figure 1). This review is divided in three sections aiming to revise the absorption of major red wine flavonoids, their metabolism and influence of gut microbiota (Topic 1), followed by the discussion of their interaction with biological proteins (Topic 2) and revisiting some of the health-promoting effects of red wine reported so far (Topic 3).

Future works should take into consideration several key points. Indeed, it is necessary to use foods or chemically well characterized extracts since this has been the major drawback of most of the epidemiological studies and human trials, making difficult to clearly comprehend any positive results or to establish any structure-activity relationships. Furthermore, in vitro assays, namely concerning the interaction with biological proteins as well as modulation of cell signaling pathways, should be done with the red wine flavonoid metabolites found in vivo. Most of the studies carried out used native unmodified forms of flavonoids found in red wine and hence should be interpreted with caution.

## 2. Bioavailability of Red Wine Phenolics

### 2.1. Absorption and Metabolism of Red Wine Anthocyanins and Derivatives

Anthocyanins are water soluble pigments found in plants, flowers and fruits, making them naturally consumed in the human diet [18]. The beneficial effect of red wine has been attributed to the presence of polyphenolic compounds including anthocyanins. Although several reports have addressed the biological properties of anthocyanins in different biological systems, many of these studies have tested the compounds in their native forms and at concentrations far from those in the physiological range [19,20,21]. Natural anthocyanins have been reported to have some of the lowest bioavailabilities of all the dietary flavonoid subclasses, below 1% of the ingested amount, so any significant contribution of anthocyanins to health-protective properties of red wine after consumption by healthy volunteers is likely to be questionable [22,23].

In this context, the study of anthocyanin bioavailability has special relevance to drive research towards more accurate findings. The key difference compared to other flavonoid glycosides is that anthocyanins undergo rearrangements in response to pH and temperature [24].

Besides the structural features of this flavonoid class, several other physiological events may contribute to increase or decrease their plasma levels. Upstream of gastrointestinal absorption, a variety of binding processes can take place, namely interaction with food proteins or with salivary proteins and digestive enzymes, as previously described [25,26,27]. Oral transformation reactions resulting from interaction of polyphenols with saliva have not been considered as an important factor due to the short time that most foods remain in the oral cavity.

Recently, several natural black raspberry anthocyanins were detected in the mouth of healthy volunteers. In addition, ß-glycosidase activity of whole and microflora-reduced saliva enables the deglycosylation of black raspberry anthocyanins [28]. Further, the aglycone forms were intracellularly glucuronidated after uptake by cells [28].

In a human trial with red grape or chokeberry juice the stability of anthocyanins during 5 min in the mouth was evaluated and the authors concluded that the anthocyanin structure affects their oral stability and buccal cell uptake and, therefore, the potential anthocyanin structure and concentration available for further absorption [29].

Besides the possible effect of oral transformation, it is not likely for some absorption to occur in the mouth due to the low oral residence of food and the transient passage of drinks. Therefore, some studies have been conducted to understand the fast kinetics of plasma anthocyanin appearance [30,31,32,33]. Passamonti and co-workers suggested the possible involvement of the gastric barrier to the rapid absorption of anthocyanins in plasma [34]. Recently, an in vitro gastric model was developed and showed that anthocyanins could be efficiently transported in this epithelium [35,36]. In this model, absorbed levels of anthocyanins reached around 10% of the apical amount after 3 h of incubation.

Some previous studies evaluated the bioavailability of anthocyanins using red wine and dealcoholized red wine [37,38]. One of the first studies is the work of Bub and co-workers who have only detected the main native anthocyanin in plasma and urine with no effect of ethanol on the amount quantified [37]. Increases in plasma of malvidin-3-*O*-glucoside (Mv3glc) concentrations were not significantly different after the consumption of either red wine or dealcoholized red wine [37].

In a human trial study in which a grape anthocyanins extract in a sugar-sweetened yogurt was consumed, the main pigment detected in plasma was native Mv3glc followed by peonidin-3-*O*-glucoside (Pn3glc), but glucuronyl conjugates of malvidin and peonidin were also detected [39]. Similarly, after anthocyanin-rich grape juice consumption the most abundant native anthocyanins found in plasma and urine were malvidin and peonidin and glucuronidated metabolites [40].

In another work, several anthocyanin conjugates (methylated, glucuronidated and sulphated) were detected both in plasma and urine after ingestion of blackberry purees, with or without ethanol, by two groups of volunteers with different body mass indexes [41]. On the basis of the rapid appearance of the conjugates (15 min after ingestion), it was suggested that anthocyanins could be metabolized in the upper gastrointestinal tract. However, the majority of these compounds may originate in the liver and kidneys since the plasma concentration of total anthocyanin conjugates continued to increase after 60 min [41]. Moreover, this study indicated for the first time that ethanol enhances cyanidin-3-*O*-glucoside (Cy3glc) metabolism potentiating its conversion into methylated and glucuronidated derivatives (Me-Cy-Glucr and 3′-Me-Cy3glc). This effect was more pronounced in overweight and obese individuals. These results should prompt the attention of the scientific community to the fact that the kinetics of these compounds is influenced by ethanol and body mass index.

The identity and concentration of anthocyanin metabolites that are the most relevant forms in vivo are practically unknown because of the lack of standards. It is only possible to determine the type of conjugation, but it is not possible to ascertain the position of the conjugation. Since this lack of information will compromise the evaluation of conjugate bioactivity (several isomers may be assayed) and ascertaining the metabolic mechanisms followed by anthocyanins after absorption (according to our knowledge there is one main route, but secondary ones are also observed), this is a major problem.

Previously, some enzymatic and chemical hemi-synthesis strategies were developed in order to obtain the standards for metabolites that are likely to be detected in vivo after consumption of foodstuffs containing anthocyanins [42,43,44]. Glucoside forms were used to obtain metabolites since aglycones are not likely to occur in vivo due to their instability.

Following the chemical synthesis strategy of cyanidin-4′-*O*-methyl-3-glucoside and cyanidin-7-*O*-glucuronyl-3-glucoside [42], the synthesis of one probable red wine anthocyanin conjugate was performed [45].

The synthesis of radiolabeled anthocyanins makes the task of searching for anthocyanin genuine forms, metabolites or catabolites easier. According to Czank et al. anthocyanins are more bioavailable than previously reported [46]. The accumulation of multiple phenolic metabolites might ultimately be responsible for reported anthocyanin bioactivity, with the gut microflora apparently playing an important role in the biotransformation process. Nevertheless, phase II conjugates of cyanidin-3-*O*-glucoside and cyanidin (cyanidin-glucuronide, methyl cyanidin, and methyl-cyanidin-glucuronide) were also detected in plasma and urine.

The most important metabolites corresponded to products of anthocyanin degradation (i.e., benzoic, phenylacetic and phenylpropenoic acids, phenolic aldehydes, and hippuric acid) and their phase II conjugates, which were found at 60- and 45-fold higher concentrations than their parent compounds in urine and plasma, respectively [46].

All in all, this data suggests that anthocyanins would be as bioavailable as other flavonoid subclasses, such as flavan-3-ols and flavones, which have relative bioavailabilities between 2.5% and 18.5% [23].

Chemical reactions of polyphenols are particularly important in wine because they are responsible for the color and taste changes that occur during aging. Grape polyphenols, namely, anthocyanins, flavonols, hydroxycinnamic acids, and flavanols including catechins and proanthocyanidins, represent approximately one half of the polyphenol content of a 2 year old red wine polyphenol extract [47]. The other half consists of unknown phenolic species derived from grape polyphenol reactions during winemaking and aging. Various chemical reactions involving anthocyanins and/or flavanols have been demonstrated to occur during red wine aging [48]. Anthocyanin pyruvic-acid adducts can rapidly reach rat plasma after oral administration of malvidin-3-glucoside-pyruvic acid adduct [49]. Also, flavanol-anthocyanin pigments were found to be absorbed in the intestinal Caco-2 cell model [50].

Based on reported studies, a new, often overlooked field of interest has risen: anthocyanin-derived pigments (dimeric anthocyanins or flavonol-anthocyanin dimers) acting as “pro-anthocyanins” (delivery systems) or having their own biological impact in the organism.

Present data indicate that the formation of anthocyanin adducts with pyruvic acid, decreases hydroxyl and superoxide anion scavenging and thus could decrease the antioxidant potential of these compounds [51]. Recently, several pyranomalvidin-3-glucosides were suggested as good antioxidant compounds because they easily donate an H atom to the free radicals. The originating stable species [52] supported the fact that the antioxidant potential arising from anthocyanins is not impaired by some of their transformations during red wine aging.

Considering the biological activity of those anthocyanins derivatives, only few in vitro studies have been performed demonstrating that anthocyanin-pyruvic acid adducts possess anticancer properties by inhibiting breast cancer cell proliferation and by acting as cell antiinvasive factors and chemoinhibitors [53].

### 2.2. Absorption of Flavan-3-ols

Although a recent in vitro study using a human cell model found that around 2% of procyanidin dimers could cross a gastric barrier after 180 min [54], the stomach is not generally considered an absorptive organ. The possible formation of procyanidin-salivary protein complexes [55] in the mouth may contribute to reduced gastric absorption in in vivo conditions.

Both human and animal studies indicated that (+)-catechin and (−)-epicatechin were rapidly absorbed from the upper portion of the small intestine. Maximum (+)-catechin levels in human plasma were reached 1.4 h after the intake of dealcoholized red wine [56]. Many cell, animal, and human studies have shown that flavanol monomers, are also extensively metabolized to *O*-methylated forms and/or conjugated to glucuronides and sulphates during absorption into the circulation [57,58,59]. One such metabolite, 3′-*O*-methylepicatechin, has been shown to exert protective effects against oxidative stress-induced cell death [60].

Major conjugates of (−)-epicatechin in human plasma, bile, and urine were (−)-epicatechin 3′-*O*-sulfonate and (−)-epicatechin 3′-*O*-β-glucuronide after ingestion of 50 mg of (−)-epicatechin by volunteers [61]. Conjugation of (−)-epicatechin with glucuronic acid had already been observed in human blood brain barrier cell line model [62].

Proanthocyanidins are not likely to pass the lipid bilayer via the transcellular pathway due to their large number of hydrophilic hydroxyl groups [63]. Paracellular diffusion was thought to be a preferential absorption mechanism since similar absorption percentages were observed for (+)-catechin and procyanidin dimers and trimers [64]. Oligomers were found to be transported through Caco-2 cell monolayer although with low absorption rates [65].

There is some controversy regarding whether polymeric proanthocyanidins can be cleaved into monomeric units during the passage through the gastrointestinal tract. The cleavage of higher procyanidin oligomers to mixtures of monomers and dimers in the stomach may act to enhance their absorption in the small intestine as higher oligomers have very limited absorption [66]. A later study suggested that procyanidin dimers and trimers were highly stable under gastric and duodenal digestion conditions [67] and other studies reported that procyanidins with a degree of polymerization less or equal than three are absorbable [68,69,70]. In addition, a recent study did not report any (−)-epicatechin ((−)-EC) in human plasma and urine after ingestion of procyanidin oligomers and polymers [71].

Current knowledge indicates that proanthocyanidin dimers and trimers are absorbed in their intact forms and their absorption rates are less than 10% of (−)-EC. The absorbed oligomers seem to undergo less phase II metabolism than (−)-EC [63]. Conversely, unlike monomers that suffer almost complete phase II transformation into glucuronides, sulphates and methylethers, procyanidin B2 3-*O*-gallate was partially absorbed intact when administrated orally to mice with no significant metabolites detected in plasma [72].

Likewise, some human studies have detected unconjugated procyanidin B1, B2, and B5 in plasma and serum within 30 min after consumption of the test material [73,74]. Unconjugated procyanidin B2 concentration in the systemic circulation reaches a peak at approximately 2 h after administration in humans [73,74]. However, the levels of intact procyanidin B2 detected in human plasma (~10–40 nM) after ingestion of high doses of proanthocyanidins are lower, sometimes by several orders of magnitude, than the concentrations observed to be effective in various in vitro tests. After oral consumption by mice of 250 mg per kg body weight of grape seed extract containing monomers and procyanidins B1, B2, B3, B4, C1, the urine samples were found to contain flavan-3-ols and their methyl derivatives. The detection of proanthocyanidins in urine also showed that excretion depends on the degree of polymerization. The excretion was very poor for dimeric and trimeric procyanidins, with the total amount varying between 14 and 20 μg for the dimeric procyanidins and being approximately 5 μg for procyanidin C1 [69]. Other authors have also shown that dimeric and trimeric procyanidins are present in plasma and tissues [68,70,75,76,77].

Conversely, studies in humans [71,78] showed that depolymerization in the gastrointestinal tract was insignificant, and proanthocyanidins were stable during gastric transit. As a result, the concentration of monomers in blood or urine was independent of the amounts of oligomers and polymers present. The majority of proanthocyanidins reach the colon intact and are degraded into phenylvalerolactones and phenolic acids by the colon microbiota [63,79,80,81]. Some of the products of this degradation may have greater biological activity than their parent compounds [82] and may be later absorbed by enterohepatic recirculation and then suffer phase II metabolism in the liver.

### 2.3. Microbiota Impact on Red Wine Phenolic Bioavailability

Since the first stage of wine consumption is in the oral cavity, the processing of wine components by oral microbiota and the modulation of a diversity of bacteria by these components could be relevant. According to some recent data, the overall diversity and stability of representative bacterial groups of the human saliva is not disturbed by regular to moderate red wine consumption [83].

Wine components that reach the gut microbiota are those not absorbed in the upper GI level as well as their metabolites that are excreted in the bile and/or from the enterohepatic circulation.

Red wine polyphenols are extensively metabolized by microbiota especially by specific genera and species with enzymes able to catalyze these compounds (hydrolyzing and conjugating enzymes) [84,85,86]. Therefore, colon is being considered as an active site for metabolism rather than a simple excretion route and has been receiving much attention from the scientific community [87].

These transformations may contribute to the absorption and modulation of the biological activity of red wine phenolics, which is different between metabolized and natural forms [88,89].

In a human intervention study, modulation of gut microbiota using red wine was already described as an effective strategy for managing metabolic diseases associated with obesity [90].

An intervention study with red wine revealed significant changes in eight metabolites: 3,5-dihydroxybenzoic acid, 3-*O*-methylgallic acid, *p*-coumaric acid, phenylpropionic acid, protocatechuic acid, vanillic acid, syringic acid, and 4-hydroxy-5-(phenyl)valeric acid [91], without any effect of ethanol on the microbial action. It is important to highlight that *O*-methylbenzoic acids such as syringic and vanillic acids could arise from methoxylated anthocyanin catabolism [92,93] and protocatechuic acid from cyanidin. *p*-Coumaric acid may also be a product from *p*-coumaroyl-acylated anthocyanins [94]. Also, and since anthocyanin phenolic acids can be further absorbed in colon [92], it is possible that these phenolic acids are additionally metabolized by hepatic cells [95]. Health benefits associated with red wine anthocyanin intake may also be explained by a slow and continuous release of phenolic compounds through the gut into the bloodstream.

The total faecal metabolome contains information on the metabolites found in the intestine, from which knowledge about the metabolic function of the gut microbiota can be obtained. The metabolome characterization of human faeces after moderate consumption of red wine by healthy subjects completely identified 20 metabolites including not only the wine compounds and microbial-derived metabolites of wine polyphenols (referred above), but also endogenous metabolites and/or others derived from other nutrient pathways. After wine consumption, faecal metabolome is usually enriched in flavan-3-ols metabolites. Also of relevance was the down regulation of xanthine and bilirubin-derived metabolites such as urobilinogen and stercobilin, which may be related to an inhibitory effect on the activity of intestinal microorganisms [96].

Regular consumption of red wine (mean = 100 mL/day) appears to be associated with a reduced serum lipid peroxidation in which the intestinal microbiota may be involved [97].

Similarly to what was reported for anthocyanins, also proanthocyanidins can be metabolized by microbial enzymes. Twenty-four ‘dimeric’ metabolites with a molecular weight greater than 290 were detected after procyanidin B2 was incubated with human fecal microflora [79]. Microflora may also cleave the interflavan bond to convert procyanidin B2 into two (−)-epicatechin after procyanidin B2 incubation with human fecal flora for up to 12 h [79]. The majority of proanthocyanidins reaches the colon intact and is degraded into phenylvalerolactones and phenolic acids by colon microbiota [79,98].

In vivo studies showed that microbial derived phenylvalerolactone and phenolic acids were the predominant metabolites of procyanidins in blood and urine [71,99]. Urinary excretion of *p*-coumaric acid, vanillic acid, 3-hydroxybenzoic acid, and ferulic acid increased by over 2-fold in humans after consumption of 40 g of procyanidin-rich cocoa powder [99]. There was an increase in urinary excretion of microbial catabolites after human consumption of procyanidins and catechin monomers [100].

Recent studies unraveled the pivotal role of gut microbiota on human health [101]. Increasing evidences suggest that anthocyanins and proanthocyanidins have the potential to confer health benefits via modulation of the gut microbiota and by exerting prebiotic-like effects [102,103,104].

Thinking about the first contact of the original molecules with the upper gastrointestinal tract some important features need to be explored, especially those related to the interaction with macromolecules.

## 3. Polyphenol Interactions with Biological Proteins

### 3.1. Polyphenols Inside the Oral Cavity and Oral Health Promotion

It is well-known that red wine polyphenol interaction with some salivary proteins (SP) is particularly important. This interaction, especially with salivary proline-rich proteins (PRPs) (acidic, basic and glycosylated) and statherins yield (in)soluble aggregates, which is majorly related to astringency sensation. Astringency is a tactile sensation described as dryness, tightening and puckering sensations perceived in the oral cavity, well-known for red wine consumers and highly associated with red wine quality. In general, the nature of tannin/protein interactions is mainly non-covalent through hydrophobic and hydrogen bonds although some covalent interactions could also occur.

Among polyphenols, tannins are the major compounds known to interact with SP as well as with other proteins. However, an interaction was recently reported between the major red wine anthocyanin (malvidin-3-glucoside) with salivary acidic PRP [105] as well as with anthocyanin-derived pigments, namely pyranoanthocyanins.

The interaction of polyphenols with SP is quite significant because the oral cavity is the first point of contact between the human body and red wine polyphenols. It has been found that some polyphenols (procyanidins) and PRPs form stable complexes in the oral cavity, which remain stable throughout the whole gastrointestinal tract, being resistant to stomach digestion. By this way, polyphenols are not released from complexes to be further metabolized and absorbed. This impairs bioavailability and the further biological effects of polyphenols.

Besides this interaction with SP, polyphenols are also known for their health-promoting actions inside the oral cavity. In fact, oral cavity is the human body location where polyphenols are in highest concentration compared to all other tissues. When we refer to oral health, a major concern is dental caries. At present there is very active research to identify functional foods and their components that are generally recognized as safe with the aim of developing natural approaches for the reduction or prevention of caries. In this field of research, a number of foods showed potential anticaries activity including red grape seeds and red wine (proanthocyanidins) [106,107,108]. An extensive review on this field was done recently by Abachi and co-workers [109]. In addition to exerting antibacterial activity against *Streptococcus mutans* adhesion to saliva-coated hydroxyapatite (sHA) (bacteria responsible for caries), proanthocyanidins also promote *S. mutans* detachment from sHA [107]. Wine was also found to strongly inhibit biofilm formation. The main responsible substances for these activities were found to be procyanidins. It was also demonstrated that red wine could inhibit ex vivo *Streptococcus mutans* biofilm formation on the surface of extracted human teeth [107,110]. Besides procyanidin effects on biofilm and cariogenic virulence factors of oral streptococci, there are several studies on the inhibition of glucosyltransferase enzymes, responsible for the production of insoluble bio-adhesive polysaccharides, forming a plaque that mediates firm adherence of oral streptococci to the dental surface. Grape phenolic extracts obtained from *Vitis vinifera* varieties Cabernet Franc and Pinot Noir were all able to remarkably inhibit glucosyltransferases B and C [111].

Besides caries prevention, proanthocyanidins from grape seeds (an extract rich in oligomers) are also known to reduce cell proliferation in human oral cancer cells infected by human papillomavirus, implicated in the development of some oral cancers, through cytotoxic activity and inducing apoptosis [112,113].

### 3.2. Interaction with Enzymes

Besides SP, the interaction of polyphenols with other biological proteins, namely enzymes, is of high importance. From digestive enzymes to enzymes involved in cell proliferation, the scope of interactions and actions of polyphenols is quite diverse. In fact, the anticancer activity of several polyphenols is due to their ability to inhibit enzymes involved with carcinogenesis and tumor development, as detailed bellow [114,115].

#### 3.2.1. Effects on Carbohydrates Metabolism and Interaction with Glucosidases

Regarding the digestive enzymes, several polyphenols such as flavonoids (anthocyanins, catechins, flavanones, flavonols, flavones) and tannins (proanthocyanidins and ellagitannins) have been reported to interact and inhibit several digestive enzymes [116,117].

Potential efficacy of polyphenols on carbohydrate metabolism and glucose homeostasis has been well investigated both in vitro, animal models and some clinical trials [118,119,120,121]. Amylases and α-glucosidase are the key enzymes responsible for digestion of dietary carbohydrates into glucose. These enzymes are present in the oral cavity and are also secreted by the pancreas to the small intestine, being responsible for the digestion of carbohydrates. Disorders of carbohydrate metabolism may cause severe health problems such as diabetes, obesity, or dental caries. So, these enzymes are drug design targets in attempts to treat the referred diseases.

**Figure 2:**
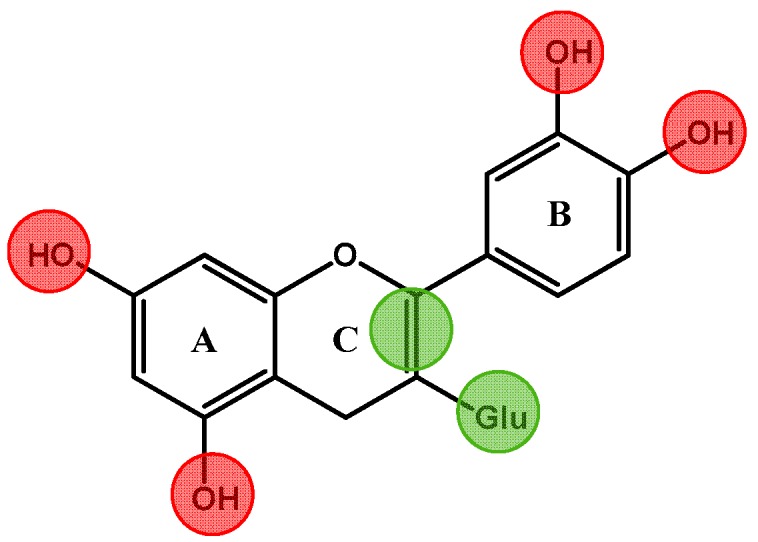
General structure of flavonoids highlightening the effect of the substitution pattern responsible for increasing (red) or decreasing (green) the inhibition of digestive enzymes. Glu stands for glycosylation.

It has been well reported that polyphenols have the ability to inhibit these enzymes. An interesting review summarized several flavonoid structural features that affect their inhibitory capacity on these enzymes [118] (Figure 2). The hydroxylation on rings A and B of flavonoids improved the inhibition against these digestive enzymes. In fact, the hydroxylation at positions C-3′ and C-4′ of B-ring of flavonoids remarkably improved the inhibition while the glycosylation of hydroxyl group at C-3, weakened the inhibition. Regarding other flavonoid substitutions at ring B such as methylation, the effects depended on the enzyme tested.

Regarding condensed tannins, results in the literature concerning their effect on digestive enzyme activities is not consensual. Barret and co-workers found that larger and more complex proanthocyanidins (trimers and tetramers) isolated from fruits (such as grapes) inhibited amylase and glucosidase more effectively than less polymerized tannins (monomers) [122]. However, Lee and co-workers found that the effect of proanthocyanidin polymerization degree is dependent on the enzyme [116]. These authors observed that polymers showed a strong inhibitory activity against α-amylase, while oligomers had a relatively weak effect. On the other hand, oligomers exerted a stronger protective effect than polymers against α-glucosidase activity. Gonçalves and co-workers observed the same trend for amylase inhibition [123]. The most polymerized procyanidins (from pentamers to hexamers) were more effective inhibitors of amylase activity than less polymerized structures (trimers). Yamashita and colleagues also found that high polymerization degree procyanidins (mDP ≥ 4) were more effective in inhibiting the α-glucosidase activity [124]. These authors observed that the anti-hyperglycemic effect of procyanidins was due to low-DP procyanidins (mDP ≤ 3) that mainly contribute to stimulate glucose uptake by glucose transporter 4 translocation through AMPK-dependent pathway in skeletal muscle while high mDP procyanidins contribute mainly to the inhibition of α-glucosidase activity in small intestine.

Adisakwattana and colleagues found that grape seed tannins, besides inhibiting intestinal pancreatic α-amylase and α-glucosidase activities, were also able to inhibit the process of glycation which results in delayed carbohydrate digestion of absorbable monosaccharide [125].

Altogether, polyphenols could in theory slow starch digestion by inhibition of these digestive enzymes but could also exert other important biological modulations related to carbohydrate metabolism increasing satiety by modulation of glucose “spiking” and depletion that occurs after carbohydrate-rich meals. This could make the use of these natural compounds as antidiabetic agents possible.

#### 3.2.2. Effects on Lipid Metabolism and Interaction with Lipases

Polyphenols have also been reported to affect lipid metabolism being strongly linked to weight loss, obesity and cardiovascular protection. Several mechanisms have been reported including reduction of dietary lipid digestion and absorption from the gut. Once again, this inhibition could occur by interaction and inhibition of lipase, a digestive enzyme.

Before fat can be absorbed, triglycerides from foods must be hydrolyzed and pancreatic lipase plays a key role in the efficient digestion of these compounds. Grape seed extracts and oligomeric procyanidins (higher than trimer) inhibit the activity of pancreatic lipase, thus limiting dietary triglyceride absorption [117,126,127,128]. Inhibitory effects of procyanidins increased from dimeric to pentameric procyanidins with pentamer or higher DP procyanidins showing maximal inhibitory effects on pancreatic lipase. Kurihara and collaborators also found that small structural modifications in polyphenols lead to pronounced differences in lipase activity inhibition [129]. While epicatechin gallate, a common flavan-3-ol present in red wine, significantly inhibits lipase, epigallocatechin does not. Also, Sbarra and colleagues found that cholesterol esterase from humans and rats, an enzyme involved in the duodenal digestion of lipids, was inhibited by a red wine polyphenol extract [130]. The same enzyme was also inhibited by a grape seed extract [128].

Other authors observed several benefits related to lipid metabolism on high fat diet rats using grape seed extract rich in procyanidins, namely normalized plasma triglyceride and LDL-cholesterol, decreased hypercholesterolemia and fatty liver, decreased body weight gain, including reduced adiposity index [131,132]. Bladé and collaborators made an extensive revision comprising in vivo studies on the proanthocyanidin effects on lipid metabolism [133].

Besides lipase and cholesterol esterase, other authors suggest that the hypolipidemic effects of procyanidins are related to inhibition of other key enzymes in lipid biosynthesis pathways. Ardévol and colleagues found that procyanidin extracts from grape and wine inhibited glycerol-3-phosphate dehydrogenase activity, an enzyme essential in adipocyte synthesis of lipids [134]. Once again, the extract efficiency was related to the procyanidins mean DP.

Procyanidins also have the capacity to interact directly with lipids reducing their availability. The micellar solubility of cholesterol in vitro is more significantly decreased by procyanidin dimers B2 and B5, trimer C1, and tetramer A2 than by catechin and epicatechin, suggesting that procyanidins could inhibit cholesterol and bile acid absorption by decreasing micellar cholesterol solubility [135].

#### 3.2.3. Effects on Cells Signaling Pathways and Interaction with Kinases

Besides digestive enzymes, polyphenols’ bioactivities may be related to more complex effects related to cell signaling through significant interaction with signaling proteins and enzymes. For instance, several interactions have been identified for quercetin, a flavonoid present in red wine. Quercetin has the potential to bind to the ATP-binding sites of a large number of proteins, including mitochondrial ATPase and protein kinase C, among others [136,137,138,139,140]. These interactions could modulate several cellular mechanisms since protein kinase C enzymes play important roles in several signal transduction cascades. Quercetin may also interact directly with mitochondria, for instance by modulating the mitochondrial transition pore which controls cytochrome C release during apoptosis [137,141].

Other noteworthy interactions are with target enzymes for anticancer drug development. DNA topoisomerase II poisons represent some of the most important and widely prescribed anticancer drugs currently in clinical use. These drugs stabilize DNA-topoisomerase complexes that will ultimately trigger cell death. Several years ago, tannins, including gallo-, ellagi-, condensed, and complex tannins, usually present in significant concentrations in red wine, were found to be inhibitors of human DNA topoisomerase II in vitro [142]. Relative inhibitory activity was primarily related to the number of phenolic hydroxyl groups (galloyl moieties) found in the active structures, with more groups generally conferring increased effect. More recent studies have observed the same trend [143]. An interesting study has focused on polyphenol structural elements that rules the mechanism by which polyphenols interact with topoisomerase II [144]. EGCG and EGC have been proved to be redox-dependent topoisomerase II poisons, kaempferol and quercetin were traditional poisons and myricetin used both mechanisms. On the basis of their findings, the authors proposed that C4′-OH of B ring is critical for the compound to act as a traditional poison, the addition of –OH groups at C3′ and C5′ increases the redox activity of the B ring and allows the compound to act as a redox-dependent poison. The second rule centers on the C ring. The structure of the C ring in flavonols is aromatic and planar and includes a C4-keto group that allows the formation of a proposed pseudo ring with the C5-OH. Disruption of these elements abolishes enzyme binding and stops the ability to function as a traditional topoisomerase II poison. Myricetin cytotoxic effects caused by inhibition of topoisomerase I has also been previously reported [145]. To a more or less extent, all the referred polyphenols have been reported to be present in red wine, although some of them are present in low concentrations.

However, most of these studies focus on the effect of each compound alone and the possibility of synergic effects are generally not considered. An interesting study that took synergy into account was made by Jo and colleagues [146]. These authors studied mixed polyphenolic fractions (anthocyanin-rich; catechins, procyanidin dimers, and flavanone-rich; and procyanidin oligomers and polymer-rich fractions) and observed that each alone effectively inhibited topoisomerase, but when mixed they showed additive effects toward catalytic inhibition.

An interesting commentary to Lim paper [147] titled this topic as “Sphingosine kinase: a key to solving the ‘French Paradox’?” [148]. Another study that used a grapevine extract evidenced that green tea and wine polyphenols impair prostate cancer cell growth in vitro and in vivo by inhibiting the sphingosine kinase 1/sphingosine phosphate pathway [114].

Another enzyme that has been associated with cancer is ornithine decarboxylase. High levels of this enzyme have been proven to be an increased risk factor and are associated with skin, breast and prostate cancers. It was observed that a polyphenolic fraction isolated from grape seeds was able to inhibit epidermal ornithine decarboxylase activity in mice when used before 12-*O*-tetradecanoylphorbol-13-acetate [115]. Other authors have shown that (−)-epicatechin, (+)-catechin, and dimers of both gallocatechin and epigallocatechin types, as well as a series of oligomeric proanthocyanidins were able to inhibit this enzyme [149].

Another attempt to explain the French paradox at a molecular level was made by Rosenkranz and co-workers [150]. This and other articles have observed the inhibitory action of red wine polyphenols on platelet-derived growth factor receptor, that are cell surface tyrosine kinase receptors for the platelet-derived growth factor, which plays a critical role in the pathogenesis of atherosclerosis [6].

### 3.3. Interaction with Serum Proteins and Platelets

Besides the interaction and inhibition of enzymes, there are several studies about the interaction of wine polyphenols with plasma proteins, which could also affect delivery of polyphenols and their metabolites to cells and tissues. The reversible binding of polyphenols to blood serum proteins such as serum albumin, α1-acid glycoprotein and lipoproteins has been reported [151].

Several authors have reported the interaction of human serum albumin and similar interactions have been reported for other proteins with several red wine polyphenols, namely quercetin [152], catechin, epicatechin, epicatechin gallate and procyanidin dimers B3 and B4 [153,154]. It was found that the interaction of some compounds with albumin weaken polyphenol antioxidant capacity, namely for gallic acid, epigallocatechin as well as catechin and epicatechin [155]. Similarly, Riedl and co-workers found that the rate of radical scavenging by procyanidin dimer B1 was decreased in the presence of protein (model albumin and gelatin) because procyanidin B1 and the proteins formed substantial amounts of perceptible complexes [156]. A similar trend was observed for quercetin bound to serum albumin [157]. These authors showed that the quercetin-albumin interaction decreased the total antioxidant activity in comparison to an equivalent amount of free quercetin.

Another interesting work focused on identifying which principal plasma proteins in humans and rats bind to red wine catechins and procyanidins ex vivo [158]. Human and rat plasma and serum were incubated with (+)-catechin and procyanidins from grape seed and these authors found that apo A-I in humans and transferrin in rats were the main bound proteins. The fact that red wine procyanidins bind to both proteins suggests that they may have a role in reverse cholesterol transport and in oxidizing iron.

Several red wine polyphenols inhibit platelet aggregation [1,159]. Platelets are important blood elements because they are active in the initiation of thrombosis as well as the development of atherosclerosis. It is also well known that patients with diabetes, hypertension, and smokers have hyperactive blood platelets. Therefore, this natural red wine polyphenol activity to reduce platelet aggregation is of great importance in preventing atherosclerosis. Beretz and co-workers found that quercetin and catechin, as well as other natural occurring polyphenols, inhibited platelet aggregation [160]. These authors studied a wide library of compounds and identified several structural features of polyphenols that diminished this effect, such as saturation of the C-2, C-3 double bond, lack of the C-4 carbonyl, glycosylation at C-3 and a high number of hydroxyl substituents.

Ruf and colleagues were able to reproduce the protective effect of red wine that they previously observed on platelets by condensed tannins (procyanidins) extracted from grape seeds or red wine, and added to 6% ethanol [161].

### 3.4. Interaction with Neurotoxic Proteins

A very important neuroprotective activity has been attributed to some red wine polyphenols, namely neuroprotection due to interaction with proteins in the brain. With an emphasis on neurodegenerative conditions such as Alzheimer’s disease, there are some polyphenols capable of directly interfering with the Alzheimer’s disease hallmark β-amyloid protein (Aβ), thereby inhibiting fibril and aggregate formation [162,163].

For example, gallic acid and catechin-rich grape seed polyphenolic extract inhibited cognitive deterioration coincident with reduced levels of soluble high molecular weight oligomers of Aβ [163].

Several mechanisms suggested the neuroprotection action of polyphenols. Some important research has been focused on polyphenols that reduce Aβ plaque pathology by inhibiting amyloid aggregation and fibrillization either as a result of metal chelation activity or by favoring the formation of nontoxic oligomers. Additional mechanisms have been also suggested through the inhibition of β-secretase (BACE-1) and/or activation of α-secretase (ADAM10). An extensive review of all these mechanisms was done by Vauzour [164].

Some research that studied the effect of two unrelated red wines, a Cabernet Sauvignon and a Muscadine wine, observed that Cabernet Sauvignon wine was effective in reducing the generation of Aβ peptides, while polyphenols from the Muscadine wine attenuate Aβ aggregation [165,166,167]. The authors identified anthocyanin compounds as the responsible for Cabernet Sauvignon effect and regarding the Muscadine wine, they pointed out that its polyphenolic components significantly interfere with Aβ protein-to-protein interaction that is critical for the initial assembly of monomeric Aβ peptides into increasingly large aggregated species.

The same authors found that grape seed polyphenolic extracts enriched with proanthocyanidins are also capable of interfering with tau-mediated toxicity by interfering with the abnormal aggregation of tau [168,169].

With the goal of understanding the action of polyphenols on Tau protein at a molecular level that could explain their beneficial effects on illness progression, Guéroux and collaborators studied the mode of action on Tau protein aggregation of ten different flavan-3-ols including several red wine polyphenols (epicatechin, epigallocatechin, procyanidin dimers B1 to B4, the C4–C8 trimer of catechin and the B3 galloylated dimers) using NMR and dynamic molecular modelling [170]. The results showed that the fixation to protein occurs in the peptide region where phosphorylations usually take place and the affinity has been shown to depend on the presence of some procyanidin structural elements (the presence of a galloyl moiety and a higher degree of polymerization were shown to increase the affinity).

### 3.5. Interaction with Allergy Proteins and Other Antimicrobial Activities

Another possible biological action of polyphenols is related to anti-allergic effect. The interaction of polyphenols with proteins can modulate the process of allergic sensitization and their direct effect on allergic symptoms. The ability of some phenolic acids such as caffeic, ferulic and gallic acid to form insoluble complexes with potentially allergenic proteins can render the protein hypoallergenic [171,172,173].

The antimicrobial activity of polyphenols is useful against infectious diseases. For example, the alleged anti-HIV activity could be due to inhibition of enzymes, such as reverse transcriptase, proteinase and integrase, and inhibition of CD4 receptors. Polyphenols’ activity against human and avian influenza viruses appears to be mainly due to the inhibition of viral haemagglutinin, while the activity against cytomegalovirus is attributed to inhibition of epidermal growth factor receptors and immediate early protein function [174,175].

## 4. Wine Flavonoids in Health and Disease Prevention

### 4.1. Cardiovascular Protection

The consumption of alcohol can have beneficial or harmful effects, depending on the amount consumed and the profile of the consumer. Some epidemiological studies have found that moderate alcohol consumption is associated with a lower risk of cardiovascular disease (CVD) and a reduced risk of all-cause mortality among middle-aged and older adults. In addition, moderate alcohol consumption may also be associated with better cognitive function [176]. Red wine bioactives have been associated with cardiovascular mortality rate reduction in France in comparison with other countries that have a similar consumption of saturated fats, the so-called “French paradox”. The cardiovascular protection of wine flavonoids has been described as the result of multiple biological activities including improvement of endothelial functionality, decrease of Low Density Lipoprotein (LDL) uptake, diminution of LDL oxidation and aggregation, reduction of blood pressure and inhibition of platelet aggregation. Wine flavonoids are thought to protect against the disease by exerting hypocholesterolemic effects. Hypercholesterolemia is well established as a risk factor of atherosclerosis. Two independent studies on healthy subjects and smokers showed no change in the levels of triglyceride, LDL, High Density Lipoprotein (HDL) and total cholesterol after red wine consumption [5].

Inflammation is recognized as a key process in atherogenesis [177]. Flavonoid mechanisms of action as anti-inflammatory compounds are unclear. A potential anti-inflammatory action is the ability to inhibit the biosynthesis of eicosanoids. Some flavonoids have been shown to inhibit both cyclooxygenase (COX) [178] and 5-lipooxygenase (5-LO) pathways [179]. Free arachidonic acid is metabolised by these two enzymes to generate the prostanoids and leukotrienes which are the first active proinflammation compounds, starting the whole process of inflammation. A recent study verified that wine flavonoids switch off the nuclear factor kappa-light-chain-enhancer of activated B cells (NF-κB) pathway, thus reducing the release of pro-inflammatory cytokines [177]. NO release following activation of extracellular regulated kinase and p38 kinase is able to inhibit platelet aggregation, thus reducing the influx of atherogenic monocytes and LDL through endothelial cells into the wall of arteries and ultimately limiting atherogenesis. Furthermore, NO production may reduce infectious events, such as *Chlamydia pneumoniae* infection, which represents an important etiological factor in atherosclerosis [180]. As far as the role of lipopolysaccharide (LPS) in atherogenesis is concerned, inhibition of phosphorylated p38 expression in the presence of LPS, as well as the regulatory role of nuclear factor of kappa light polypeptide gene enhancer in B-cells inhibitor, alpha (Iκba) mediated by wine flavonoids, should be taken into consideration in the arrest of the proinflammatory cytokine pathway in a variety of diseases. To date, major studies were focused on the endothelial origin of NO as a regulator of vascular homeostasis. However, in a recent study different wines were screened for modulating nitric oxide (NO) production from monocytes [177]. Platelet activity and thrombosis inhibition were also verified [181]. The exact mechanisms by which wine flavonoids inhibit platelet activity are not yet fully understood but it is possible that flavonoid effects changes in membrane fluidity, ligand-receptor affinity, and intracellular signalling pathways. Flavonoids may mediate their effect through antioxidant and NO-related pathways [182].

### 4.2. Flavonoids as Cancer Chemopreventive Compounds

Several aspects of the Mediterranean diet, namely moderate wine consumption, are related with protective effects (or reduced incidence) not only on cardiovascular diseases but also on several cancers. Although numerous anticancer therapies are currently in clinical use, mortality as well as new cancer cases are still alarmingly high. With progress in understanding the molecular biology of cancer, it is now evident that cellular homeostasis is disrupted. Many of these altered cell signaling pathways can be reversed and restored to a normal state by chemopreventive compounds such as flavonoids. Different mechanisms of flavonoids acting as carcinopreventive agents have been described [179,180,181,182,183]. Polyphenols can modulate the action either of proteins in charge of their extracellular export and of enzymes that detoxify carcinogens [183], such as the platelet-derived growth factor receptor (for example, several anthocyanin) [16]. Zell and colleagues found that regular wine consumption had favorable effects in colorectal cancer cases not only on disease stage at presentation but also for survival [184]. Protection of moderate wine consumption was also detected against renal cancer in a meta-analysis [185]. The findings of several case-control studies provided support for a protective role of flavonoids in different gastrointestinal cancers. In vitro and clinical studies have shown that red wine inhibits the proliferation of cells from different human cancers—such as lung, oral squamous carcinoma and prostate [186,187]. In a meta-analysis for the effect of subclasses of dietary flavonoids by cancer type, flavanones were related to the upper aerodigestive tract [7], flavonols and anthocyanins to the colorectal [15], flavonols and flavones to breast cancers [188].

### 4.3. Wine and Obesity

Obesity and related conditions are currently explained by a linear view of disease progression, in which expansion of white adipose tissue (WAT) generates a proinflammatory microenvironment that spreads through organs and tissues, hindering their function and causing the devastating comorbidities associated with obesity. This model can be refined by adding genetic predispositions and factors linked to lifestyle such as diet or lack of exercise that influence the onset and progression of obesity and obesity-related conditions. In this way, obesity, although modulated by genetic predispositions, could be considered as an “acquired” environmental disease to be treated by targeting causative elements such as defective or excessive nutrition or sedentary lifestyle. However, in recent years, the concept of obesity as an “epigenetic disease” has begun to be discussed. Nutrition can affect the expression of a number of genes by its impact. Since food is potentially important for the development of “metabolic memory”, there is a need for more information on the type of nutrients causing adverse or toxic as well as positive effects. Thus, a growing number of epigenetically active compounds are currently tested in clinical trials for their therapeutic potential. Interestingly, many phytochemicals present in plant foods and beverages such as wine, particularly flavonoids, are suggested to be able to alter epigenetic cellular mechanisms [189]. The role of flavonoids in obesity is based on mechanisms that reduce food intake/absorption, promotes energy expenditure, or prevents energy storage. One of the popular approaches to weight loss is appetite control. The food urge and satiety are controlled by serotonin, histamine, dopamine and their receptors [190]. Besides food intake reduction through neuroreceptor control there are other mechanisms related to obesity dealing with lipid oxidation. Stimulated energy expenditure can be used to reduce body weight by induction of non-shivering thermogenesis. Thermogenesis mechanisms activate several uncoupling proteins found in the mitochondria of brown adipose tissue (BAT). The uncoupling proteins (UCP), namely also thermogenins, mediated heat generation at BAT allowing for fast lipid oxidation with a low rate of ATP production. In lipid metabolism, peroxisome proliferator-activated receptor gamma (PPAR-γ) and 5′-adenosine monophosphate-activated protein kinase (AMPK) also play crucial roles. PPAR-γ is a transcriptional factor (mediating gene expression) predominately expressed in adipose tissue that stimulates adipose differentiation. Therefore, PPAR-γ agonists can ameliorate dyslipidemia (lipoprotein overproduction or deficiency), as well as improve adiposity and insulin resistance. AMPK is an enzyme which regulates the target proteins controlling metabolism. AMPK activation regulates glucose transport and fatty acid oxidation. Recent studies have demonstrated that many dietary polyphenols as mimic caloric restriction and activate AMPK, thus suppressing hepatic gluconeogenesis, inducing hepatic fatty-acid β-oxidation, and stimulating glucose transporters in muscle and adipose tissues, with an overall reduction of the glucose level in the blood and the lipid content of the liver as well as an improvement in insulin sensitivity [191,192].

From all of the proposed mechanisms, one of the most promising approaches to weight management is the decrease in fat absorption. As aforementioned, flavonoids can interact with digestive enzymes [193]. Flavonoids could interact with lipases interfering therefore in fat catabolism.

### 4.4. Diabetes Mellitus

Diabetes, a group of metabolic diseases characterized by high blood sugar, is an important worldwide health issue. An estimated 346 million people worldwide have type 2-diabetes according to the World Health Organization (WHO), with this number projected to double by 2030. Type 2 diabetes is affected by lifestyle. Several studies have proposed the relationship between diets rich in fruits and vegetables and type 2-diabetes prevalence [121]. Poor glycemic control associated to type 2-diabetes could therefore be mitigated by diet. Therapy for diabetes mellitus aims to obtain an optimal blood glucose level following a meal. Since α-glucosidase and α-amylase both play significant roles in controlling insulin, inhibition of these enzymes has become a therapeutic goal in order to prevent glucose absorption, and, ultimately, suppress postprandial hyperglycemia. Several in vitro studies verified that red wine exhibits strong inhibitory activities both on α-glucosidase, which catalyses the cleavage of glucose from disaccharide and α-amylase, which breaks down long-chain carbohydrates [194,195]. Wine flavonoids also inhibit glucose absorption in the intestine by sodium-dependent glucose transporter 1 (SGLT1) [119,196,197], which stimulates insulin secretion and reduces hepatic glucose output. As discussed about obesity mechanisms, flavonoids could activate AMPK [198]. This phenomenon was found to improve insulin sensitivity in the skeletal muscle and liver of type 2 diabetic mice [199]. The AMPK activation was often accompanied by the up-regulation of glucose transporter 4 GLUT4 in skeletal muscle and the down-regulation of gluconeogenesis in the liver in type 2 diabetic mice [199]. However, human epidemiological and intervention studies have shown inconsistent results. In a randomized controlled study, red wine significantly improved insulin sensitivity, lipid profiles and endothelial function comparatively to other alcoholic beverages [200]. In a recent study [201], ten male volunteers randomly consumed one of four tested beverage of red wine, vodka, beer (0.32 g ethanol/kg body weight) and water (control). Every beverage was consumed once a week in a cross-over design. The volunteers breathed 100% normobaric O_2_ between 60th and 90th min of 3-h study protocol. Plasma lipid peroxides (LOOH—biochemical marker of oxidative stress) and uric acid (UA) concentration, blood alcohol concentration (BAC) and arterial stiffness—indicated by augmentation index (AIx)—were measured before and 30, 60, 90, 120 and 180 min after beverage consumption [201]. Moreover, 2 g/day of grape seed extract (1 g of polyphenols) also improved endothelial function in subjects with high vascular risk [202]. Overall, there is little evidence of an effect of wine polyphenols on glucose and insulin. Further intervention studies are essential to clarify the conflicting findings and confirm or refute the anti-diabetic effects of dietary polyphenols.

### 4.5. Neurological Diseases

Fruit and vegetable consumption may be important to neuronal ‘health’. The current understanding of the metabolism of flavonoids and potential modes of protection in neuronal cell systems, the current status of the understanding of flavonoids as neuroprotective agents in vivo will be considered in this section.

Neuroinflammation constitutes a beneficial process involved in the maintenance of organ homeostasis and the brain response to infection or injury [203]. However, sustained neuroinflammatory processes may contribute to the cascade of events leading to the progressive neuronal damage observed in ageing [204] and aged-related cognitive disorders such as Parkinson’s disease [205] and Alzheimer’s disease [206,207]. Brain damage and neuroinflammatory process begin years before substantial neurodegeneration appears. Hence, there is a crucial need for the development of new strategies capable to prevent, delay the onset or treat brain dysfunction and associated cognitive decline. Growing evidence sheds light on the use of dietary polyphenols to improve cognitive performance and reduce the neuroinflammatory and oxidative stress responses occurring with age and neurodegenerative pathologies. Indeed, evidence has emerged from human intervention trials that demonstrate consumption of flavonoid-rich foods is associated with cognitive benefits [208,209,210]. Inflammation has been discussed as an important contributor to neuronal damage in neurodegenerative disorders [211,212] and is known to involve the generation of both ROS and reactive nitrogen species (RNS). NO and superoxide (O_2_˙^−^) may be also generated in the brain and their formation is often co-localized in specific neurons. According to Ishige and coworkers [213] using mouse hippocampal cell line HT-22 and primary cortical neurons, flavonoids could protect cells from oxidative damage by three distinct mechanisms: increasing intracellular glutathione, directly lowering levels of ROS and preventing the influx of Ca^2+^. They also suggested that certain structural requirements were necessary to mediate such properties including a hydroxylated C3, an unsaturated C ring and lipophilicity. Furthermore, other in vitro and in vivo studies have indicated that the flavanones hesperetin, naringenin and their relevant in vivo metabolites, as well as some dietary anthocyanins, cyanidin-3-rutinoside and pelargonidin-3-glucoside, are able to cross the BBB [214,215,216]. These findings also suggested that the potential for flavonoid penetration is dependent on compound lipophilicity [214]. Unfortunately, the findings from these various studies aimed at identifying mechanisms of neuroprotection generally employ the use of flavonoids in their native form and not those that are likely to enter the circulation. Despite finding intact anthocyanins in the cortex and cerebellum [217,218], several absorption studies verified that anthocyanins appeared in the plasma and urine mainly as a glucuronidated conjugate [216,219]. Recent studies suggested that both, anthocyanins and their main metabolites could interact with microglia biology and furthermore with neuron biology [220]. Although further work is necessary to establish their bioavailability to the brain, particularly in human subjects, these results suggest that flavonoids and their main metabolites may localize in the brain and are capable of direct or indirectly affecting neuroprotective and neuromodulatory actions.

The impact of flavonoids on peripheral and cerebral blood flow reinforces the importance of understanding to what extent cognitive improvements are mediated by circulating metabolites in the periphery or/and directly by flavonoid metabolites within the brain. Acute studies have shown that several flavonoid food sources [221,222,223,224,225,226] resulted in working memory improvements and further reduced depressive symptoms in both adult and even in children [227,228] after flavonoid-rich diet supplementation. These studies revealed some remarkable differences in comparison to the vehicle [229]. On the other hand, the use of cognitive tests that are sensitive enough to detect effects of dietary interventions on human cognitive function is critical to fully assess the efficacy of flavonoid-rich foods in humans [230,231]. Despite some particular benefits of anthocyanin supplementation, some other long-term effects may not be desirable and further studies are needed to optimize ingestion conditions. Overall and despite some encouraging cognitive outcomes emerging from human clinical trials, further studies are required to address these questions with adequate sample sizes and better characterization of the test materials in relation to their flavonoid content, as well as appropriate controls that are matched for all other micronutrients and macronutrients.

In the specific case of wine consumption, there is a controversy between the flavonoid and the alcohol content. The effect of moderate alcohol intake on neurological diseases is unclear. A prospective study in The Netherlands observed that the consumption of red wine, but no other type of alcoholic beverages, was inversely associated with the progression of cognitive decline in a population of middle age subjects. These findings support the hypothesis that non-alcoholic substances found in red wine are involved in a favorable effect on cognitive function [232,233]. There is some experimental support for these clinical observations. Whereas some authors [234,235,236] established some associations between moderate wine consumption (3–4 glasses/day) and a reduced incidence of dementia and Alzheimer’s disease compared with non-drinkers, some others argue the contrary [237,238]. Reports have also shown that heavy drinkers displayed the poorest results on memory or on intelligence tasks when compared with moderate or non-drinkers, suggesting that the effects of sustained and excessive alcohol consumption cannot be negated by potential positive influences of wine flavonoids [239,240,241,242].

Some investigators have claimed that the urinary excretion of isoprostanes, a class of eicosanoids derived from fatty acid oxidation in arachidonic acid, meets enough reliability for measuring oxidative stress in vivo [243]. The antioxidant effect of red and white wine was investigated in healthy volunteers by measuring urinary content of prostaglandin-F2-III (PR-III), an indicator of the isoprostane production, after 15 days of supplementation. A greater decrease in urinary neuropeptide PF2 was observed in those who were given red wine as compared to white wine, which was attributed to the higher polyphenol content of red wine. Of interest, dealcoholized wine was capable of maintaining antioxidant benefits, supporting the role of polyphenols in oxidative stress production as evaluated by isoprostane concentrations.

## 5. Conclusions

Wine, and in particular red wine, has been often reported to be positively related to health-promoting properties. This evidence has been associated with the ingestion of several flavonoids present in large amounts in red wine. However, the concentration required to trigger a biological event is dependent not only on the amount ingested, but also on critical variables that include bioaccessibility, bioavailability, stability under in vivo conditions, etc. On the other hand, local accumulation in target tissues or organs may also be a relevant issue for chronic consumption and should not be overlooked. Therefore, a clear vision on the real bioavailability of wine flavonoids is difficult to achieve as there are several pieces of the puzzle that have to be collected and carefully set up together to unravel this matter. Many studies are still required to clarify many of these issues related to the health-promoting properties of wine.
